# A Dual Nanosensor Approach to Determine the Cytosolic Concentration of ATP in Astrocytes

**DOI:** 10.3389/fncel.2020.565921

**Published:** 2020-09-18

**Authors:** Susanne Köhler, Hartmut Schmidt, Paula Fülle, Johannes Hirrlinger, Ulrike Winkler

**Affiliations:** ^1^Carl-Ludwig-Institute for Physiology, Faculty of Medicine, University Leipzig, Leipzig, Germany; ^2^Wilhelm-Ostwald-Schule, Gymnasium der Stadt Leipzig, Leipzig, Germany; ^3^Department of Neurogenetics, Max-Planck-Institute for Experimental Medicine, Göttingen, Germany

**Keywords:** ATeam, ATP, astrocyte, calibration, genetically encoded sensors for metabolites

## Abstract

Adenosine triphosphate (ATP) is the central energy carrier of all cells and knowledge on the dynamics of the concentration of ATP ([ATP]) provides important insights into the energetic state of a cell. Several genetically encoded fluorescent nanosensors for ATP were developed, which allow following the cytosolic [ATP] at high spatial and temporal resolution using fluorescence microscopy. However, to calibrate the fluorescent signal to [ATP] has remained challenging. To estimate basal cytosolic [ATP] ([ATP]_0_) in astrocytes, we here took advantage of two ATP nanosensors of the ATeam-family (ATeam1.03; ATeam1.03YEMK) with different affinities for ATP. Altering [ATP] by external stimuli resulted in characteristic pairs of signal changes of both nanosensors, which depend on [ATP]_0_. Using this dual nanosensor strategy and epifluorescence microscopy, [ATP]_0_ was estimated to be around 1.5 mM in primary cultures of cortical astrocytes from mice. Furthermore, in astrocytes in acutely isolated cortical slices from mice expressing both nanosensors after stereotactic injection of AAV-vectors, 2-photon microscopy revealed [ATP]_0_ of 0.7 mM to 1.3 mM. Finally, the change in [ATP] induced in the cytosol of cultured cortical astrocytes by application of azide, glutamate, and an increased extracellular concentration of K^+^ were calculated as −0.50 mM, −0.16 mM, and 0.07 mM, respectively. In summary, the dual nanosensor approach adds another option for determining the concentration of [ATP] to the increasing toolbox of fluorescent nanosensors for metabolites. This approach can also be applied to other metabolites when two sensors with different binding properties are available.

## Introduction

The metabolism of the brain is realized by a joint effort of all cell types including neurons, glial cells as well as cells constituting the blood vessels. Almost all aspects of metabolism in the brain involve several types of cells, including energy metabolism (Pellerin and Magistretti, 1994; Guzmán and Blázquez, 2001; Barros and Deitmer, 2010; Nave, 2010a; Hirrlinger and Nave, 2014; Barros et al., 2018b; Díaz-García and Yellen, 2019; Vicente-Gutierrez et al., 2019; Zuend et al., 2020), and neurotransmitter metabolism (van den Berg et al., 1978; Waagepetersen et al., 2003; Bak et al., 2006; Le Douce et al., 2020). Furthermore, brain cells are structurally intermingled, heavily interdigitating their numerous cellular processes (Somjen, 1988; Grosche et al., 1999; Bushong et al., 2002; Nave, 2010b). Therefore, to study the dynamics of brain metabolism in detail, methods allowing to assess metabolites at high spatial and temporal resolution are required. These requirements are not fulfilled by most of the classical biochemical methods, as well as by techniques like PET or fMRI.

However, fluorescence nanosensors for metabolites are well suited to follow the concentration of a growing set of metabolites at high spatial and temporal resolution *in vitro*, *in situ*, and *in vivo* (Imamura et al., 2009; Hung et al., 2011; San Martin et al., 2014; Lange et al., 2015; Mächler et al., 2016; Mongeon et al., 2016; Trevisiol et al., 2017; Winkler et al., 2017; Köhler et al., 2018; Barros et al., 2018a; Díaz-García et al., 2019; Zuend et al., 2020). Conceptually, these sensors are proteins composed of a domain which specifically binds the metabolite of interest as well as one or more fluorescent proteins, which change their fluorescent properties upon metabolite binding. Different types of such sensors have been described. In single fluorophore sensors the fluorophore changes its fluorescence intensity upon metabolite binding. In ratiometric sensors the signal change is either derived from Förster resonance energy transfer (FRET) involving two fluorescent proteins. Alternatively, a second “reference fluorophore” is integrated in the sensor protein, which is unaffected by metabolite binding. Examples of such sensors include sensors for adenosine triphosphate (ATP; e.g., sensors of the ATeam family, Imamura et al., 2009; Queen, Yaginuma et al., 2014; Takaine et al., 2019), the ATP/ADP ratio (Perceval, Berg et al., 2009; PercevalHR, Nguyen et al., 2019), the NADH/NAD^+^-redox ratio (Peredox, Hung et al., 2011; Sonar, Zhao et al., 2015), NADPH (iNap family, Zhao et al., 2016), glucose (FlipGlu, Fehr et al., 2003; SweetieTS, Díaz-García et al., 2019), lactate (Laconic, San Martín et al., 2013), and pyruvate (Pyronic, San Martín et al., 2014; PyronicSF, Arce-Molina et al., 2020).

Fluorescent nanosensors for metabolites can be used to monitor relative changes of the respective metabolite within a cell or cellular compartment during an experiment by monitoring the time course of the fluorescent signal. However, the fluorescence signal is often not linearly related to the concentration of the metabolite (or only in a small concentration range). Furthermore, all sensors show a basal, metabolite independent fluorescence. Therefore, to calculate the concentration of the metabolite from this fluorescence signal has remained challenging and several different approaches have been employed (San Martin et al., 2014; Yellen and Mongeon, 2015; Barros et al., 2018a). All of these different approaches have their own advantages, but also limitations (see discussion section and Table 1). We here add a novel approach to the toolbox of fluorescent nanosensors for metabolites, which allows estimating the intracellular concentration of a metabolite under basal conditions by combining two nanosensors for the same metabolite with different binding equilibrium constants. This approach is exemplified using two ATP sensitive nanosensors (ATeam1.03, abbreviated AT, and ATeam1.03YEMK, abbreviated ATY) which differ in their dissociation constants (k_D_; Imamura et al., 2009). The feasibility of this approach is shown in cultured cortical astrocytes as well as in cortical astrocytes *in situ* in acutely isolated brain slices.

**TABLE 1:** Comparison of the dual nanosensor approach to other methods for calibrating nanosensors.

	**Dual nanosensor**	**Single point calibration**	**Full calibration**
Requirements	2 Sensors with different k_D_	1 Sensor	Precise control of the [metabolite] within the cellular compartment of interest, e.g., via - transporters - permeabilization - dialysis
Parameters to be obtained from other experimental systems	k_D_ n_H_ (for both sensors)	k_D_ n_H_ R_max_	None
Parameters to be measured in each experiment^1^	R_min_	R_min_	None^2^
Single cell data available	Yes^3^	Yes	Yes

*Notes: ^1^in addition to R. ^2^If the signal is very stable between experiments. However, at least one “anchor point” (e.g., R_0_) might be necessary to relate the established calibration curve to the specific experiment. ^3^needs second step of calculation.*

## Materials and Methods

### Ethics Statement

In accordance with the guidelines for the welfare of experimental animals issued by the European Communities Council Directive (2010/63/EU) and with the German Protection of Animals Act (Tierschutzgesetz), mice were bred in the animal facility of the Medical Faculty of the University of Leipzig. Mice were housed in individually ventilated cages in a specific pathogen free environment in a 12 h/12 h light dark cycle with access to food and water *ad libitum*. Experiments were approved by the animal welfare office of the Faculty of Medicine, University of Leipzig and the governmental authorities of Saxony (Landesdirektion Sachsen, registration number T20/16; TVV62/15).

### Cell Culture and Transfection

Plasmids pDR-GW AT1.03, pDR-GW AT1.03YEMK, and pDR-GW AT1.03R122K/R126K (Bermejo et al., 2010) were obtained from Wolf Frommer (via Addgene; plasmids 28003, 28004, and 28005). The open reading frames of ATeam1.03, ATeam1.03YEMK, and AT1.03^*R*122K/R126K^ (Imamura et al., 2009) were subcloned into pDEST expression vectors using Gateway cloning. Primary cortical astrocytes were prepared from the brains of newborn mice of the C57Bl/6J background as described (Requardt et al., 2010, 2012; Winkler et al., 2017). Cells were seeded on glass coverslips (30 mm diameter) with custom made silicon rings, which allow splitting the surface of the coverslip into two independent chambers (Supplementary Figure 1), and were cultured in DMEM/10% FCS/25 mM glucose for 1 week. The medium was exchanged to DMEM/10% FCS/5 mM glucose and cells were further cultured for at least 7 days with exchange of the medium twice per week. Cells on one coverslip were transfected with expression plasmids encoding ATeam1.03 in one chamber and ATeam1.03YEMK in the other chamber using lipofectamine (Thermo Fisher Scientific, Schwerte, Germany) and the standard protocol suggested by the supplier. Cells were used for experiments 1 day after transfection.

### Epifluorescence Imaging

Epifluorescence live cell imaging was performed as essentially described before (Winkler et al., 2017). In brief, coverslips were mounted into a custom-made flow chamber which was continuously perfused at 37°C with incubation medium containing (in mM) 145 NaCl, 5.4 KCl, 1 MgCl_2_, 1.8 CaCl_2_, 20 HEPES, 0.8 Na_2_HPO_4_, 5 glucose; pH 7.4; bubbled with 20% O_2_, 80% N_2_ to ensure a constant O_2_ partial pressure in all experiments. Cells were observed using an Axio-ObserverZ1 microscope (Zeiss, Jena, Germany) using a Plan-Apochromat 20x/0.8 objective and an Axiocam 506 camera (688 × 552 pixels, 4 × 4 binning, pixel size 0.91 μm × 0.91 μm) with acquisition times of 150 ms (CFP) and 100 ms (FRET). The two channels were recorded with the following filter sets: CFP: excitation 436/25 nm, beam splitter 455 nm, and emission 480/40 nm; and FRET: excitation 436/25 nm, beam splitter 455 nm, and emission 535/30 nm. Using the motorized x-y stage of the microscope, images of cells in both chambers were recorded almost simultaneously with only the time delay necessary to move the stage to the other positions (<1 s). Images were obtained every 20 s. At the end of each experiment, iodoacetate (1 mM; Applichem, Darmstadt, Germany) and sodium azide (10 mM; Serva, Heidelberg, Germany) were applied. This treatment blocks all ATP-producing pathways allowing to normalize the sensor signal to nominally [ATP] = 0.

### AAV Injection and 2-Photon Imaging in Acute Brain Slices

To image the dynamics of ATP in cortical astrocytes in acute brain slices, ATeam1.03 or ATeam1.03YEMK-open reading frames (Imamura et al., 2009) were cloned into a vector for packaging into adeno-associated viruses (AAV) and astrocyte-specific expression was driven by GFAP-promoter elements (Lee et al., 2008; Mächler et al., 2016; Stobart et al., 2018). The plasmids were packaged into AAV serotype 5 by the Viral Vector Facility at the Neuroscience Center Zurich, University of Zurich, Switzerland. 0.5 μl of AAV containing solution (virus titer: 2.2 × 10^12^ vg/ml (AT); 2.75 × 10^12^ vg/ml (ATY) were stereotactically injected into cortex of 2 to 3 month old mice (coordinates: 0.5 mm caudal to bregma, lateral 2.0 mm, and ventral 1.1 mm), i.e., in the primary somatosensory cortex. 4 weeks later, mice were sacrificed and 250 μm thick acute coronal brain slices were prepared (Pätz et al., 2018). Slices were transferred to the stage of a 2-photon laser scanning microscope (Olympus FV1000) equipped with a XLPlan N 25x/1.05 W objective (Olympus) and a Mai Tai DeepSee laser (Spectra-Physics, Darmstadt, Germany) and continuously superfused at room temperature with aCSF solution (in mM): 130 NaCl, 2.5 KCl, 1 MgCl_2_, 2 CaCl_2_ 1.25 NaH_2_PO_4_, 26 NaHCO_3_, 10 glucose, pH 7.4. The medium was continuously bubbled with carbogen. Imaging was performed within 500 μm of the site of AAV injection. The following imaging parameters were used: excitation: 810 nm; emission 510 DC XR (F33-511 OD, 266685; AHF, Germany); pixel size: 0.552 × 0.552 μm; 512 × 512 pixels; pixel dwell time: 2 μs; time resolution: 60 s; stack of 23 single *z*-planes. All experiments were started by perfusing the slice with aCSF for 20 min followed by aCSF containing 1 μM TTX (Tocris, Bristol, United Kingdom) to prevent neuronal activity and to define the sensor signals under baseline conditions. Afterward, glutamate (100 μM in aCSF) was applied for 20 min. At the end of each experiment, 1 mM iodoacetate and 10 mM azide was added to deplete the cells of ATP. All solutions were adjusted to same pH and osmolality.

### Data Analysis and Analytical Solution

**FIGURE 1:**
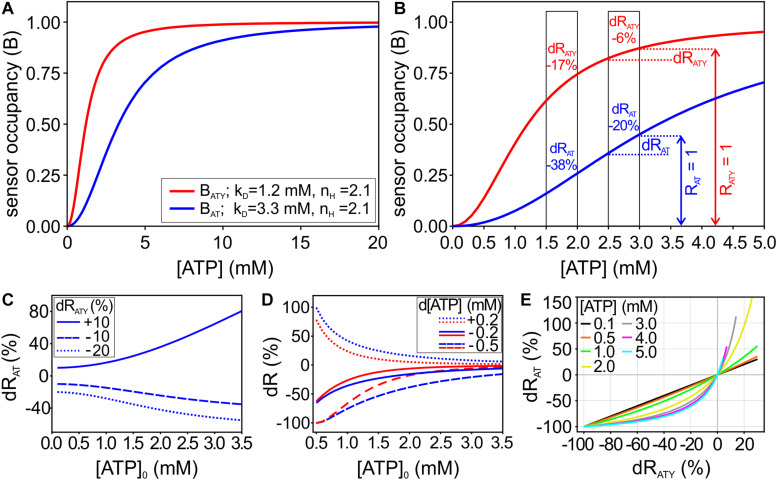
Mathematical framework of the dual nanosensor approach. **(A)** Binding curves for the ATP sensors ATeam1.03 (AT; blue) and ATeam1.03YEMK (ATY; red), showing the sensor occupancy (B) at different cytosolic [ATP]. **(B)** Enlargement of A for [ATP] from 0 to 5 mM. The boxes highlight two examples with d[ATP] = −0.5 mM starting at [ATP]_0_ = 2 mM and [ATP]_0_ = 3 mM, respectively. Note that R_AT_ = 1 and R_ATY_ = 1 (i.e., the sensor signal at the starting [ATP]_0_, indicated by the vertical arrows for the example of [ATP]_0_ = 3 mM) correspond to different values of sensor occupancy (B) for both sensors. The resulting relative changes of the sensor signals dR_AT_ and dR_ATY_ are given within the boxes. **(C)** Changes of R_AT_ (dR_AT_) depending on [ATP]_0_ at given values of dR_ATY_ (dR_ATY_ = −10%, −20%, and +10%). **(D)** A change in [ATP] results in a characteristic pair of signal changes dR_AT_ and dR_ATY_, which depend also on [ATP]_0_. Shown are examples of d[ATP] = −0.2 mM, d[ATP] = −0.5 mM, and d[ATP] = +0.2 mM. **(E)** For each [ATP]_0_, unique pairs of dR_AT_ and dR_ATY_ describe a given d[ATP]. Therefore, [ATP]_0_ can be deduced from pairs of dR_AT_ and dR_ATY_ obtained experimentally.

For a detailed description of the variables and indices used for this calculation see Supplementary Table 1. Regions of interest (ROI), each containing a single cell, were defined manually using Fiji (Schindelin et al., 2012). Background subtracted mean fluorescence intensities averaged over all pixels within a ROI were determined for the FRET (I_FRET_) and the CFP (I_CFP_) channel. The ratio of the intensities (*I*) was calculated as I = I_FRET_/I_CFP_. The relative sensor signal ratio (R) was calculated as

(1) $$R=\frac{I-I_{m\,i\,n}}{I_{0}-I_{m\,i\,n}}$$

where I_min_ is I at [ATP] of 0 mM and I_0_ being the pre-treatment baseline I at the unknown baseline [ATP]_0_. This results in a minimum R (R_min_) of 0 for [ATP] of 0 mM and a pre-treatment *R* value (R_0_) of 1 at the unknown baseline [ATP]_0_. Notably, R as defined by Eq. 1, does not directly relate to the sensor occupancy B, defined as

(2) $$B=\frac{\left[A\,T\,P\,b\,o\,u\,n\,d\,s\,e\,n\,s\,o\,r\right]}{{\left[s\,e\,n\,s\,o\,r\right]}_{t\,o\,t\,a\,l}}=\frac{{\left[A\,T\,P\right]}^{n_{H}}}{{\left[A\,T\,P\right]}^{n_{H}}+k_{D}^{n_{H}}}$$

with n_H_ being the Hill-coefficient and k_D_ the dissociation constant. B scales between 0 (at [ATP] = 0) and 1 (at full saturation of the sensor; Figures 1A,B). However, while at [ATP] = 0 both, B and R are equal to 0, R = 1 at [ATP]_0_ for each experiment but *B* = 1 at full saturation of the sensor (Figures 1A,B). As [ATP]_0_ within a cell is not known, the value of *B*_0_ at baseline conditions is also not known; therefore, a direct calculation of changes in [ATP] (d[ATP]) from changes in R (dR) is not possible.

If *R* values at full saturation (R_max_) can be measured, it is possible to relate R to the metabolite concentration, as has been done for the ATeam-type ATP sensors using purified sensor proteins (Imamura et al., 2009):

(3) $$R=\frac{R_{m\,a\,x}\times {\left[A\,T\,P\right]}^{n_{H}}}{{\left[A\,T\,P\right]}^{n_{H}}+k_{D}^{n_{H}}}$$

Different from this R_max_ approach, the dual nanosensor approach introduced here avoids the necessity to determine R_max_. Yet, it still relies on knowledge about k_D_ and n_H_ (Table 1). To calculate [ATP]_0_ and [ATP] during treatment ([ATP]_treat_), dR values for measurements with AT and ATY, respectively, were obtained experimentally for different treatments; k_D_ and n_H_ values for the indicators were taken from the literature. The following sets of parameters were used:

k_D,AT_ = 3.3 mM, k_D,ATY_ = 1.2 mM, n_H,AT_ = n_H,ATY_ = 2.1 (Imamura et al., 2009).

k_D,AT_ = 9.4 mM, k_D,ATY_ = 2.7 mM, n_H,AT_ = n_H,ATY_ = 1.0 (Lerchundi et al., 2020).

To calculate [ATP]_0_ and [ATP]_treat_ from dR_AT_ and dR_ATY_ measured in the experiments, a system of two non-linear equations was used which allowed deriving analytical solutions:

(4a) $$d\,R_{A\,T}=\frac{R_{A\,T,t\,r\,e\,a\,t}}{R_{A\,T,0}}-1$$

(4b) $$d\,R_{A\,T\,Y}=\frac{R_{A\,T\,Y,t\,r\,e\,a\,t}}{R_{A\,T\,Y,0}}-1$$

Introducing R according to Eq. 3 into Eqs 4a,b the following equations result (for simplicity “n_H_” is replaced by “n” in Eqs 5a,b and 6a,b):

(5a) $$d\,R_{A\,T}=\left(\frac{R_{m\,a\,x,A\,T}\times {\left[A\,T\,P\right]}_{t\,r\,e\,a\,t}^{n}}{{\left[A\,T\,P\right]}_{t\,r\,e\,a\,t}^{n}+k_{D,A\,T}^{n}}\right)/\left(\frac{R_{m\,a\,x,A\,T}\times {\left[A\,T\,P\right]}_{0}^{n}}{{\left[A\,T\,P\right]}_{0}^{n}+k_{D,A\,T}^{n}}\right)-1$$

(5b) $$d\,R_{A\,T\,Y}=\left(\frac{R_{m\,a\,x,A\,T\,Y}\times {\left[A\,T\,P\right]}_{t\,r\,e\,a\,t}^{n}}{{\left[A\,T\,P\right]}_{t\,r\,e\,a\,t}^{n}+k_{D,A\,T\,Y}^{n}}\right)/\left(\frac{R_{m\,a\,x,A\,T\,Y}\times {\left[A\,T\,P\right]}_{0}^{n}}{{\left[A\,T\,P\right]}_{0}^{n}+k_{D,A\,T\,Y}^{n}}\right)-1$$

Solving Eqs 5a,b as a system of non-linear equations using “Solve” of Mathematica 12.1. yielded the following analytical expressions for [ATP]_0_ and [ATP]_treat_:

(6a) $${\left[A\,T\,P\right]}_{0}={\left(\frac{\left(d\,R_{A\,T}-d\,R_{A\,T\,Y}\right)\times k_{D,A\,T}^{n}\times k_{D,A\,T\,Y}^{n}}{\begin{array}{c} d\,R_{A\,T\,Y}\times k_{D,A\,T}^{n}+d\,R_{A\,T\,Y}\times d\,R_{A\,T}\times k_{D,A\,T}^{n}\\ -d\,R_{A\,T}\times k_{D,A\,T\,Y}^{n}\\ -d\,R_{A\,T\,Y}\times d\,R_{A\,T}\times k_{D,A\,T\,Y}^{n} \end{array}}\right)}^{\frac{1}{n}}$$

(6b) $${\left[A\,T\,P\right]}_{t\,r\,e\,a\,t}={\left(\frac{\left(d\,R_{A\,T}-d\,R_{A\,T\,Y}\right)\times k_{D,A\,T}^{n}\times k_{D,A\,T\,Y}^{n}}{d\,R_{A\,T\,Y}\times k_{D,A\,T}^{n}-d\,R_{A\,T}\times k_{D,A\,T\,Y}^{n}}\right)}^{\frac{1}{n}}$$

Of note, this analytical solution requires that n_H,AT_ = n_H,ATY_, a prerequisite met by the ATeam sensors. If n_H,__1_ ≠ n_H,__2_, solving the system of non-linear equations results in expressions involving complex numbers. In this case, numerical solving of Eqs 5a,b using “NSolve” of Mathematica is preferable.

Having established the mean [ATP]_0_ of all cells observed in a given experiment according to Eq. 6a, *R* values (Eq. 1) can be correlated to sensor occupancy B. First, *B*_0_ is calculated from [ATP]_0_ using Eq. 2. Then, all *R* values are multiplied by *B*_0_ resulting in *R* values reflecting sensor occupancy with a range of R = 0 at [ATP] = 0 to R = R_max_ = 1 at saturation of the sensor. Consecutively, [ATP] for each single cell at any time point during an experiment (including [ATP]_0_) is revealed by

(7) $$\left[A\,T\,P\right]=k_{D}\times {\left(\frac{R}{R_{m\,a\,x}-R}\right)}^{\frac{1}{n_{H}}}$$

Using Eq. 7, [ATP] can be calculated for both sensors separately. Finally, rates of [ATP] changes were calculated by linear regression of [ATP] over the time period of interest.

### Data Processing and Presentation

**FIGURE 2:**
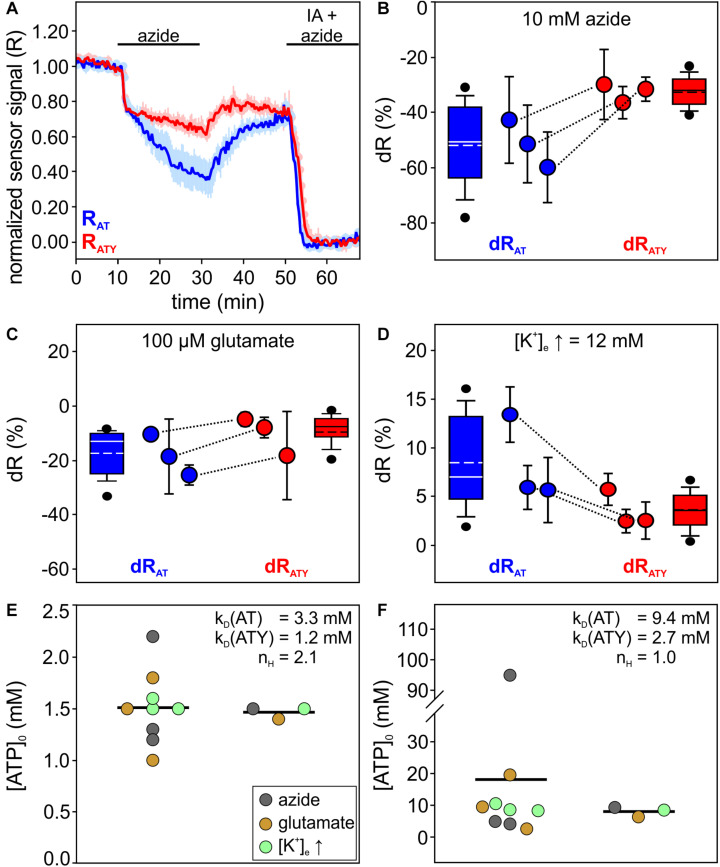
Determination of [ATP]_0_ in primary cultured cortical astrocytes using the dual nanosensor approach. **(A)** Time course of the signal of AT (blue) and ATY (red) during an experiment exposing the cells to azide (10 mM). At the end of the experiment, cells were treated with iodoacetate (IA) and azide to deplete cellular ATP. Shown is the normalized sensor signal R for all cells of one representative experiment (*n* = 25 cells for both AT and ATY; mean ± SD). **(B–D)** Signal changes dR obtained for AT (dR_AT_; blue) and ATY (dR_ATY_; red) during incubation of astrocytes with azide (**B**; 10 mM, 20 min), glutamate (**C**; 100 μM, 15 min) or an increase in [K^+^]_e_ (**D**; from 5.4 mM to 12 mM, 10 min). Shown is the distribution of dR of all cells (box plots), as well as the mean ± SD of each experiment (circles and error bars). Corresponding means of both sensors from the paired experiments are connected with lines. Each condition was replicated in *N* = 3 independent experiments. In each experiment, the following number of cells were analyzed: B, AT: 50, 50, 40. B, ATY: 48, 50, 47. C, AT: 50, 17, 40; C, ATY: 49, 44, 34. D, AT: 43, 46, 44; and D, ATY: 44, 46, 36. **(E)** [ATP]_0_ values calculated from the dR_AT_ – dR_ATY_ pairs shown in panels **(B–D)**, using the parameters indicated within the graph. On the left, values calculated from the dR_AT_ – dR_ATY_ pairs from the paired experiments (circles) as well as the mean (line) are shown. Right: values obtained from the dR_AT_ – dR_ATY_ pairs obtained from the pooled cells of all experiments. **(F)** Same calculation as in **(E)**, but with different values of k_D_ and n_H_ as indicated within the graph.

**FIGURE 3:**
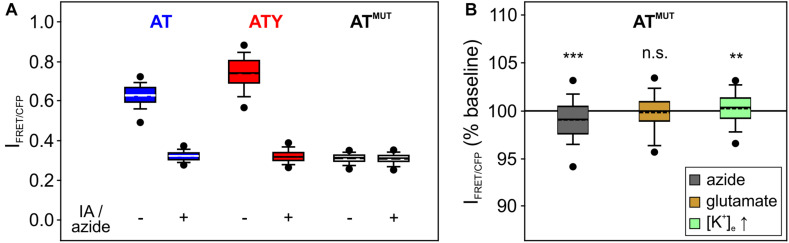
Control experiments using the ATP-binding deficient AT1.03^*R*122K/R126K^. **(A)** The signal of the ATP sensor AT and ATY during inhibition of ATP synthesis approaches the signal of AT1.03^*R*122K/R126K^ (AT^MUT^). Shown is the non-normalized intensity ratio I = I_FRET_/I_CFP_ to allow for comparison between AT, ATY, and AT^MUT^ in the absence or presence of IA + azide. The following number of cells was included in the analysis (from left to right): 381; 381; 400; 400; 810; 810 (each from *N* = 9 experiments). **(B)** The signal of AT^MUT^ is only marginally affected by application of azide, glutamate or increases in [K^+^]_e_. ***p < 0.001; **p < 0.01; and n.s. *p* > 0.05 compared to baseline, paired *t*-test. *n* = 285, 260, and 265 cells from 3 experiments each for azide, glutamate and high [K^+^], respectively.

**FIGURE 4:**
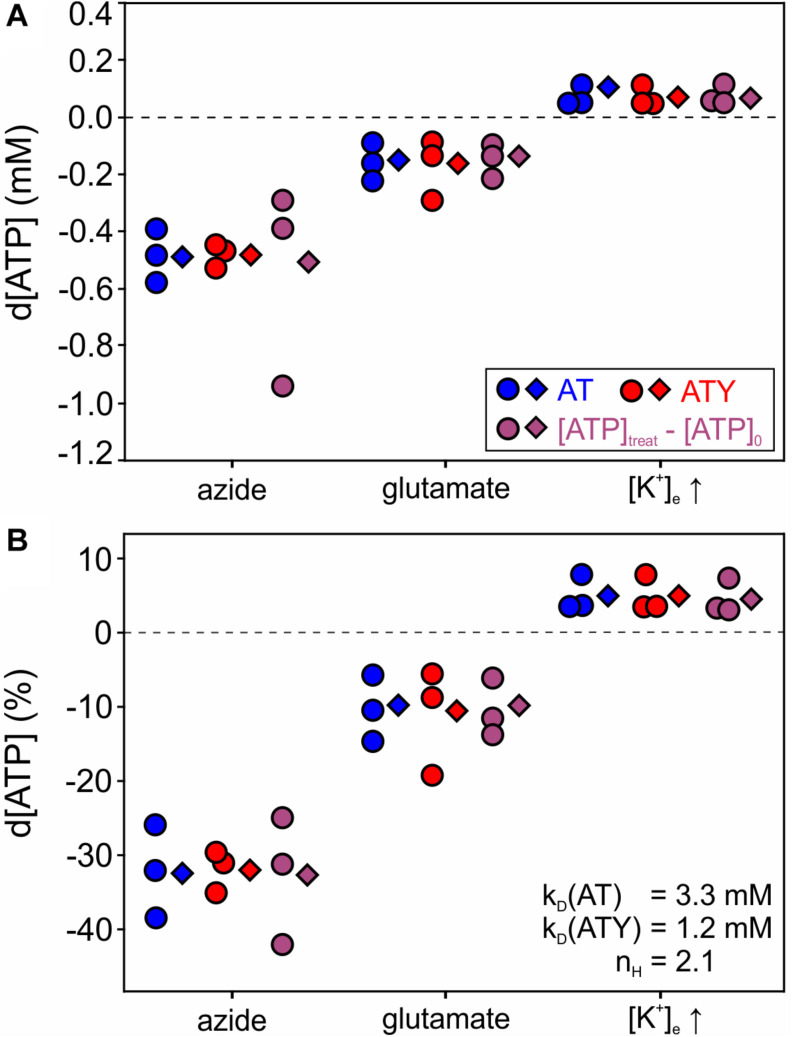
Changes in cytosolic [ATP] in astrocytes during application of azide, glutamate and increasing [K^+^]_e_. Astrocytes were incubated with azide (10 mM, 20 min), glutamate (100 μM, 15 min), or increased [K^+^]_e_ (12 mM, 10 min) and d[ATP] was calculated from nanosensor signals of AT (blue), ATY (red), or as d[ATP] = [ATP]_treat_ – [ATP]_0_ derived from Eqs 6a,b (violet). Circles represent data calculated from individual paired experiments (imaging of AT and ATY on the same coverslip in parallel), while diamonds represent calculation using pooled data from all experiments. k_D_ and n_H_ values used for this analysis are from Imamura et al. (2009) and are indicated in the figure. **(A)** d[ATP] (mM). **(B)** Same data as in A expressed as relative change (%) of [ATP]. All experiments were performed with *N* = 3 independent experiments, for number of cells in each experiment see legend of Figure 2.

**FIGURE 5:**
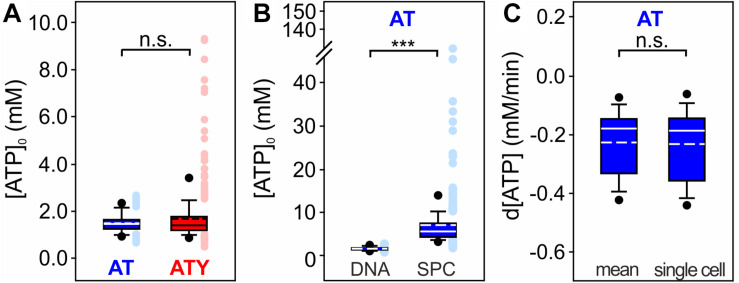
Single cell [ATP]_0_ and analysis of the dynamics of changes in [ATP]_0_. **(A)** [ATP]_0_ of each single cell calculated from the sensor signal of AT or ATY. n.s. *p* > 0.05; Mann–Whitney Rank Sum Test; *n* = 382 and 394 cells from *N* = 3 experiments for AT and ATY, respectively. **(B)** Comparison of single cell [ATP]_0_ obtained by the dual nanosensor approach (DNA) and a single point calibration protocol (SPC) assuming R_max_ = 2.3 × R_min_ (Imamura et al., 2009). The analysis was restricted to the AT sensor as no estimate for R_max_ for ATY is available. Data for the dual nanosensor approach is the same as in panel **(A)**. ***p < 0.001; Mann–Whitney Rank Sum Test; *n* = 382 and 346 cells from *N* = 3 experiments for dual nanosensor approach and single point calibration, respectively. **(C)** Rate of change of [ATP] during exposure of the cells to azide (10 mM). Shown is the slope of a linear regression for the first 1.3 min of azide application. Rates were calculated either using the mean [ATP]_0_ (mean, left box) or [ATP]_0_ calculated for each cell individually (single cell, right box); however, no significant difference was observed (*p* > 0.05; Mann–Whitney Rank Sum Test; *n* = 140 cells from *N* = 3 experiments).

Microscopic images were processed using Zeiss ZEN software, Fiji and Corel Draw X4 Graphic. Data were analyzed and calculated using Fiji and Microsoft Excel. Analytical solutions of the system of non-linear equations as well as values for [ATP]_0_ and [ATP]_treat_ according to Eqs. 6a,b were obtained using Mathematica. Diagrams were generated using Sigma Plot. In the boxplots, the box spans from the 25th to 75th percentile, the whiskers span from the 10th to the 90th percentile and dots highlighting the 5th to the 95th percentile. In addition, in Figures 5A,B all outliers are indicated by light blue and light red circles. Within the box dashed lines represent the mean value, solid lines show the median. In Figure 2A, lines represent the mean and the shaded area indicates the standard deviation (SD). In Figures 2B–D circles and associated error bars represent the mean ± SD. All data presented in Figures 1–5 are obtained on 3 independent astrocytic cell cultures. Data in the text are given as mean ± SD. Final illustrations were arranged using Corel Draw X4 Graphic.

## Results

The ATP sensors ATeam1.03 (abbreviated AT in the following) and ATeam1.03YEMK (abbreviated as ATY) are two cytosolic, ATP specific nanosensors, which differ in their affinity for ATP (Imamura et al., 2009). Dissociation constants (k_D_) of 3.3 mM and 1.2 mM were described for AT and ATY, respectively; for both sensors a Hill coefficient n_H_ of 2.1 was found (Imamura et al., 2009). Therefore, the occupancy of the sensor (i.e., the ratio of ATP bound sensor/total sensor; referred to as B here; see Eq. 2) is different for both sensors at a given cytosolic concentration of ATP ([ATP]; Figure 1A). The [ATP] of a cell under baseline resting conditions ([ATP]_0_) is a very important parameter for cellular energy metabolism, which is difficult to determine and not known in many cases. We reasoned that a treatment of cells, which results in a given change in [ATP] (d[ATP]), will cause different relative changes of the sensor signal for AT (dR_AT_) and ATY (dR_ATY_; Figure 1B). For example, at [ATP]_0_ = 2 mM a change in [ATP] of −0.5 mM will result in dR_AT_ = −38% and dR_ATY_ = −17%; while at [ATP]_0_ = 3 mM the same change in ATP will result in dR_AT_ = −20% and dR_ATY_ = −6% (Figure 1B). Therefore, at a given dR_ATY_, the corresponding dR_AT_ correlates to [ATP]_0_, or else, depending on [ATP]_0_ a change in [ATP] will result in a characteristic pair of values for dR_AT_ and dR_ATY_ (Figures 1C,D). Accordingly, [ATP]_0_ can be derived from pairs of dR_AT_ and dR_ATY_ obtained by treating cells with conditions which change [ATP] (Figure 1E). Importantly, for this approach prior knowledge of the actual d[ATP] caused by these conditions is not necessary.

To test this approach experimentally, primary cultured cortical astrocytes were studied, which have been reported before to respond to different stimuli with changes in [ATP] (Winkler et al., 2017). After transfection with either AT or ATY, cells were imaged by epifluorescence microscopy in a custom-made system allowing to image cells transfected with the different plasmids in parallel in the same experiment. Cells readily responded to treatments with a change in [ATP] reflected by both AT and ATY (Figure 2A). At the end of each experiment, cells were incubated with iodoacetate (IA) and azide to block cellular ATP production thereby depleting cellular ATP, allowing to normalize the sensor signal to the sensor signal at [ATP] = 0 mM (Figure 2A).

Cells were treated with three different conditions: (a) 10 mM azide (Figure 2B); (b) 100 μM glutamate (Figure 2C); and (c) by increasing the extracellular concentration of potassium ([K^+^]_e_) from 5.4 mM to 12 mM (Figure 2D). From the resulting pairs of dR_AT_ and dR_ATY_, [ATP]_0_ was calculated both for the single experiments with simultaneous imaging of both AT and ATY as well as for the data pooled from all experiments using the same condition (Figure 2E, Eq. 6a). Both types of analysis revealed a resting, basal [ATP]_0_ of about 1.5 mM (Figure 2E; 1.51 ± 0.35 mM, *n* = 9 experiments/1.47 ± 0.06 mM; *N* = 3 conditions).

The calculation of [ATP]_0_ using this dual nanosensor approach depends on knowledge of the k_D_ values of the two versions of the sensor. In the original description of ATP sensors of the ATeam family k_D_ values of 3.3 mM and 1.2 mM (and n_H,AT_ = n_H,ATY_ = 2.1) have been reported for AT and ATY, respectively (Imamura et al., 2009). However, based on calibration of the sensors in astrocytes and neurons in organotypic brain slice cultures, recently k_D_ values of 9.4 mM and 2.7 mM were determined (Gerkau et al., 2019; Lerchundi et al., 2020). The latter studies used a Michaelis–Menten equation for fitting the data, which inherently implies n_H_ = 1. [ATP]_0_ calculated using these parameters (Figure 2F) revealed 18.10 ± 29.23 mM (*n* = 9 experiments) and 8.04 ± 1.54 mM (*N* = 3 conditions). These values are substantially higher compared to previously published data on astrocytes and neurons (1 mM to 4 mM; Fukuda et al., 1983; Ainscow et al., 2002; Mollajew et al., 2013; Rangaraju et al., 2014; Toloe et al., 2014; Pathak et al., 2015), suggesting that this set of parameters might not be fully applicable on the experimental system used here. Despite these uncertainties regarding the k_D_ values, these analyses show that the dual nanosensor approach is well suited to estimate the intracellular cytosolic [ATP] within cells. We note, that for further analysis of the data obtained on cultured astrocytes (Figures 4, 5) the original k_D_ and n_H_ values will be used (i.e., k_D,AT_ = 3.3 mM, k_D,ATY_ = 1.2 mM, n_H,AT_ = n_H,ATY_ = 2.1; Imamura et al., 2009).

Two crucial assumptions are inherent in this approach: (a) treatment of the cells with iodoacetate + azide depletes the cell of cytosolic ATP; (b) no other factors, like, e.g., pH, affect the sensor signal during treatment of the cells. To test the validity of these assumptions, experiments were repeated with cells expressing AT1.03^*R*122K/R126K^ (abbreviated AT^M*UT*^), a mutated version of the ATeam sensors consisting of the same fluorophores and the same ATP binding protein, but which does not bind ATP due to two point mutations within the ATP binding domain (Imamura et al., 2009). First, the signal of AT^MUT^ is very similar to the signal of AT and ATY during application of iodoacetate + azide (Figure 3A), suggesting that cytosolic [ATP] reaches nominally zero and confirming previous observations (Trevisiol et al., 2017; Winkler et al., 2017). Secondly, when cells expressing AT^MUT^ were treated with azide, glutamate or with increased [K^+^]_e_, only a minor change in the AT^MUT^ signal was observed (Figure 3B; azide: −0.9 ± 2.9%; *n* = 285 cells; glutamate: −0.2 ± 2.2%; *n* = 260 cells; [K^+^]_e_: 0.2 ± 2.2%; *n* = 265 cells). As AT^MUT^ has the same properties as AT and ATY with the exception of ATP binding, these data indicate that other factors than [ATP] do not contribute substantially to the change of the sensor signal of AT and ATY under the conditions of these experiments.

Having established [ATP]_0_ using the dual nanosensor approach, the changes in [ATP] induced by the different treatments were calculated (Figure 4, Eq. 6b). Exposure of astrocytes to azide, which blocks the respiratory chain, reduced [ATP] by −0.50 ± 0.15 mM reflecting −32.4 ± 5.2% of [ATP]_0_ (Figure 4; *n* = 3 experiments with 285 cells analyzed), indicating that astrocytes maintain the cytosolic [ATP] at about 70% of the basal value in the absence of oxidative phosphorylation. Of note, vice versa this finding also implies that glycolysis alone is not sufficient to maintain [ATP] at control levels. Furthermore, application of glutamate (100 μM, 15 min) resulted in a reduction of [ATP] of about −0.16 ± 0.06 mM (−10.8 ± 5.0%; *n* = 3 experiments with 234 cells analyzed; Figure 4). Finally, increasing [K^+^]_e_ induced a rise in [ATP] of 0.07 ± 0.04 mM, i.e., 4.7 ± 2.4% (*n* = 3 experiments with 259 cells analyzed; Figure 4). The results were very similar irrespective of whether each experiment was analyzed separately, or whether all data from all cells were pooled prior to analysis (compare circles and diamonds in Figure 4), suggesting that paired experiments are not a prerequisite of the dual nanosensor approach. Furthermore, similar results were obtained when calculating d[ATP] from the AT or ATY signal (blue and red symbols in Figure 4), or by calculating d[ATP] = [ATP]_treat_ – [ATP]_0_ using the equations derived from solving the system of non-linear equations (Eqs 6a,b; violet symbols in Figure 4).

Astrocytes are a heterogenous cell population which also includes heterogeneity of metabolism (Bittner et al., 2010; Matyash and Kettenmann, 2010; Zhang and Barres, 2010; Bayraktar et al., 2015; Farmer and Murai, 2017; Köhler et al., 2018, 2019; Miller, 2018; Morel et al., 2018; Batiuk et al., 2020). Therefore, information on the heterogeneity of [ATP]_0_ within the astrocytic cell population is of major interest. While calculating [ATP]_0_ using the dual nanosensor method initially results in the mean [ATP]_0_ of all cells included in the analysis, [ATP]_0_ for each single cell can be obtained in a second calculation step using the mean [ATP]_0_ as additional parameter. This analysis revealed similar results when calculated either from the AT or ATY signals (Figure 5A; AT: 1.51 ± 0.40 mM; *n* = 382 cells from *N* = 9 experiments; ATY: 1.70 ± 1.06 mM; *n* = 394 cells from *N* = 9 experiments). However, the ATY-based calculation resulted in more cells with very high [ATP]_0_, most likely because the ATY binding curve (Figure 1A) is flat in this concentration range and even very small differences in R_ATY_ result in a rather large difference in [ATP]_0_.

Another method to obtain single cell data from fluorescent nanosensor imaging is the single point calibration protocol (e.g., Sotelo-Hitschfeld et al., 2012; Fernández-Moncada and Barros, 2014; Arce-Molina et al., 2020). Only one nanosensor is needed for this approach, and k_D_ and n_H_ are derived from other experimental systems like in the dual nanosensor approach (Table 1). In addition, an estimate for R_max_ needs to be included in the calculation. For AT, a dynamic range of R_max_ = 2.3 × R_min_ has been reported (Imamura et al., 2009). As no explicit information on R_max_ of ATY is available, only data obtained by imaging of AT were reanalyzed using the single point calibration protocol (Figure 5B). This analysis revealed a higher mean [ATP]_0_ and a larger variability of [ATP]_0_ compared to the dual nanosensor approach (Figure 5B; 7.00 ± 8.08 mM; *n* = 346 cells from *N* = 9 experiments).

Calibration of fluorescent nanosensors to the concentration of the metabolite also allows assessing kinetic changes of the concentration of the metabolite. As an example, the rate of the [ATP] decrease during inhibition of oxidative phosphorylation by azide was calculated, using either [ATP]_0_ obtained as the mean [ATP]_0_ of all cells within an experiment or [ATP]_0_ calculated for each cell individually (Figure 5C). No significant differences were observed between the two modes of calculation (*p* > 0.05; Mann–Whitney Rank Sum Test). [ATP] decreased with a rate of −0.23 ± 0.11 mM/min (Figure 5C; data calculated with mean [ATP]_0_; *n* = 140 cells from *N* = 3 experiments).

**FIGURE 6:**
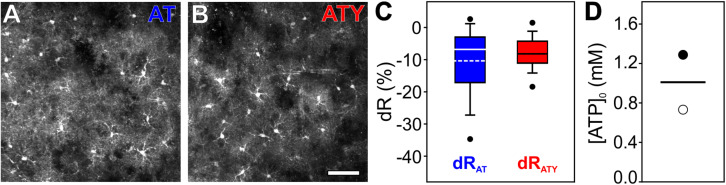
Determination of [ATP]_0_ in cortical astrocytes in acutely isolated brain slices using 2-photon imaging. AT or ATY were expressed in astrocytes in the primary somatosensory cortex *in vivo* by stereotactic injections of AAV-based vectors and acutely isolated brain slices were prepared. **(A, B)** 2-photon images showing expression of AT **(A)** or ATY **(B)** in cortical astrocytes. Scale bar: 50 μm. **(C)** dR values obtained for AT (blue) and ATY (red) during incubation of the slices with glutamate (100 μM) for 20 min. **(D)** [ATP]_0_ values obtained from the dR_AT_-dR_ATY_ pair shown in C calculated using the following sets of parameters: k_D__,AT_ = 3.3 mM, k_D__,ATY_ = 1.2 mM, n_H_ = 2.1 (open circle; Imamura et al., 2009); and k_D,AT_ = 9.4 mM, k_D,ATY_ = 2.7 mM, n_H_ = 1 (filled circle; Lerchundi et al., 2020). *n* = 80, 92 cells from N = 4, 6 mice were included in the analysis shown in **(C** and **D)** for AT and ATY, respectively.

Finally, to validate the dual nanosensor approach in a more intact system than primary cultured cortical astrocytes, AT and ATY were expressed in cortical astrocytes *in vivo* by stereotactic injection of AAV vectors, in which expression of the sensor is driven by the GFAP promoter. Acute brain slices were prepared from these mice and imaged using 2-photon microscopy (Figures 6A,B). Application of glutamate induced changes in [ATP] and corresponding values of dR_AT_ and dR_ATY_ were recorded (Figure 6C). As the two sensors were not expressed within the same mice, no pairing of experiments was possible. Based on the two different sets of k_D_-values (Imamura et al., 2009; Lerchundi et al., 2020), a basal [ATP]_0_ of 0.7 mM or 1.3 mM was calculated, respectively (Figure 6D), showing that the dual nanosensor approach can also be applied to more intact preparations like acute brain slices as well as to other imaging techniques like 2-photon laser scanning microscopy.

## Discussion

Fluorescent nanosensors for metabolites have strongly contributed to a much deeper knowledge on the metabolism and its dynamics of the mammalian brain (and of other organs and organisms; Deuschle et al., 2006; Tsuyama et al., 2013; Yaginuma et al., 2014; Masia et al., 2018; Volkenhoff et al., 2018; Nguyen et al., 2019; Takaine et al., 2019; Arce-Molina et al., 2020; Kioka et al., 2020). As these sensors are proteins which can be genetically encoded, they allow cell type specific expression using specific promoters as well as subcellular targeting using appropriate targeting sequences. Combined with different state-of-the art microscopy technologies, the dynamics of metabolites can be followed in cultured cells, in tissue preparations like brain slices or the isolated optic nerve, but also *in vivo* in living and even awake animals (Bittner et al., 2011; Ruminot et al., 2011; Mächler et al., 2016; Díaz-García et al., 2017, 2019; Trevisiol et al., 2017; Köhler et al., 2018; Baeza-Lehnert et al., 2019; Gerkau et al., 2019; Lerchundi et al., 2019; Arce-Molina et al., 2020; Zuend et al., 2020). However, while these nanosensors readily allow for monitoring relative changes of the metabolite concentration, deduction of absolute concentrations and absolute concentration changes (i.e., in mol/l) during treatments has remained challenging as calibration of the signal of the nanosensors to the actual concentration of the metabolite is hampered by both, theoretical and practical problems (Barros et al., 2018a). We here introduce the dual nanosensor approach, a novel strategy to determine [ATP]_0_ using two nanosensors for ATP with different binding equilibrium constants k_D_.

### Properties, Assumptions and Limitations of the Dual Nanosensor Approach

The dual nanosensor approach requires the availability of two nanosensors sensitive to the concentration of the metabolite of interest (here ATP), which differ in their binding equilibrium constants k_D_ (Table 1). Parallel experiments with cells expressing one or the other sensor reveal the signal change dR of both sensors (Figure 2). If both sensors would differ in their spectral properties, which is unfortunately not the case for AT and ATY (but has been reported for ATP sensors using a single fluorophore; Arai et al., 2018), both sensors could even be recorded within a single cell. Values for k_D_ and n_H_ need to be obtained from other experimental systems, e.g., from measurements of the purified sensor protein (Imamura et al., 2009), assuming that these values can be applied to the indicators in the cytosolic environment of the cells of interest (Table 1). While such transfer of parameters is often used also for other calibration strategies (e.g., Sotelo-Hitschfeld et al., 2012; Fernández-Moncada and Barros, 2014; Arce-Molina et al., 2020), several studies indicate that parameters of various genetically encoded nanosensors can be affected by the cellular environment (Hires et al., 2008; Pérez Koldenkova and Nagai, 2013; Yaginuma et al., 2014; Lerchundi et al., 2019, 2020). Moreover, care has to be taken that these values are determined by appropriate methods including similar or –ideally– identical excitation and emission wavelengths (Pomorski et al., 2013). In addition, R_min_ needs to be measured for each cell during the experiments (Table 1), thereby requiring a method to deplete the cells from ATP. Application of azide, which blocks oxidative phosphorylation at complex IV of the respiratory chain, and iodoacetate, which blocks glycolysis at glycerine-aldehyde-3-phosphate dehydrogenase (GAPDH), resulted in a decrease of the AT- and ATY-sensor signal reaching the signal of the ATP-binding deficient AT1.03^*R*122K/R126K^ (AT^MUT^; Figure 3A), suggesting that the fluorescence signal observed under these conditions is at least close to R_min_ and reflects a concentration of cytosolic ATP of nominally zero. This observation is in line with previous observations using the same treatment to deplete ATP (Trevisiol et al., 2017; Winkler et al., 2017), but also with a different approach blocking glycolysis using 2-deoxyglucose and oxidative phosphorylation by oligomycin (Shulman et al., 2015). Finally, it has to be assumed that a given treatment of a cell results in the same change of [ATP] when either AT or ATY is expressed. This might be of special importance if the concentration of the analyte of interest is within the same range (or lower) as the concentration of the sensor, e.g., in the case of Ca^2+^-sensors. However, [ATP]_0_ is at least one to two orders of magnitude higher than the typical concentration of genetically expressed sensor proteins (Barros et al., 2018a), suggesting that the difference in k_D_ of the two sensors does not have a major influence on the dynamics of [ATP].

The dual nanosensors approach adds another option to the list of strategies for quantifying metabolite concentrations from fluorescent signals. The approach eliminates the necessity to determine R_max_ (or dR_max_) of the nanosensor (Table 1) and is applicable to complex tissues like, e.g., the highly myelinated axons in the optic nerve (Trevisiol et al., 2017) or the brain *in vivo*, because it does not require experimental control of the metabolite concentration within the cell compartment of interest. Furthermore, any stimulation, which results in a reproducible change of the concentration of the metabolite, can be used to calculate [ATP]_0_ without prior knowledge of the actual concentration change d[ATP]. Finally, once the mean [ATP]_0_ of the observed cells was determined, [ATP]_0_ of each single cell can be calculated (Table 1 and Figure 5A). Therefore, this approach will be an interesting option for determining basal metabolite concentrations based on fluorescence imaging.

### Comparison to Other Methods for Calibration of Nanosensors

In the following, two other methods of calibration of metabolic nanosensors will be discussed and compared to the dual nanosensor approach: (a) single point calibration (e.g., Sotelo-Hitschfeld et al., 2012; Fernández-Moncada and Barros, 2014; Arce-Molina et al., 2020); and (b) full calibration within the cell(-compartment) of interest (used, e.g., in Bittner et al., 2010; Hung et al., 2011; San Martín et al., 2013; Mongeon et al., 2016; Köhler et al., 2018).

For calculating the metabolite concentration using the single point calibration method, k_D_ and n_H_ obtained in other experimental systems are needed and R_min_ has to be determined in each experiment (Table 1) similar to the dual nanosensor approach. While only one nanosensor is needed, additionally an estimate of R_max_ is required. However, R_max_ of, for example, the lactate sensor Laconic and the pyruvate sensor Pyronic differs by a factor of two between purified protein and when expressed in cells (San Martín et al., 2013, 2014). Therefore, a determination of R_max_ within the cell of interest is preferable, but requires experimental access to increase the concentration of the metabolite to saturating levels. Such a saturation of the sensor is feasible for metabolites for which endogenous transporters with favorable kinetic properties are expressed allowing equilibration of the extra- and intracellular concentration of the metabolite under appropriate experimental conditions, as exemplified, e.g., for glucose, lactate and pyruvate (Bittner et al., 2010; San Martín et al., 2013, 2014; Mächler et al., 2016; Arce-Molina et al., 2020). However, cells do not express transporters for ATP. Therefore, one option to increase [ATP] would be to inhibit all ATP consuming processes and to rely on cellular ATP production. However, inhibition of all ATP consuming enzymes is not feasible, and the total amount of adenine nucleotides, which can be phosphorylated to ATP, might be insufficient to achieve sensor saturation. Alternatively, cell membranes are permeabilized without leakage of the sensor protein to allow access of ATP from the extracellular environment (Gerkau et al., 2019; Lerchundi et al., 2020). However, the permeabilizing agent itself or changes of the intracellular ion composition might also affect the sensor signal. Taken together, reliable determination of R_max_ for the ATP sensor in the cell of interest is a difficult task; therefore, one advantage of the dual nanosensor approach is to avoid the need of estimating R_max_. Reevaluation of our imaging data for the AT sensor by the single point calibration method revealed mean [ATP]_0_ = 7 mM (Figure 5B), i.e., higher as calculated by the dual nanosensor approach as well as higher than previously reported for glial cells in culture (1.4 mM; Ainscow et al., 2002), suggesting that the R_max_ value obtained for the purified protein (2.3 × R_min_; Imamura et al., 2009) does not reflect R_max_ in cells. Taken together, compared to the dual nanosensor approach, the single point calibration method (Bittner et al., 2010; Mollajew et al., 2013; San Martín et al., 2013; Takaine, 2019; Arce-Molina et al., 2020) provides direct single cell information on the metabolite concentration with only a single nanosensor, but requires an additional estimate of R_max_ (Table 1).

Ideally, nanosensors are calibrated within the cell of interest at the subcellular location of interest by applying the metabolite of interest at (numerous) different defined concentrations without interfering with the cellular environment (full calibration; Table 1). Unfortunately, at present this is feasible only in a very limited set of experimental systems. First, controlling the metabolite concentration within the cell requires good accessibility to the cell of interest which is limited in more complex systems like in axons of a highly myelinated nerve or in the brain *in vivo*. In addition, access of the metabolite to the inside of the cell requires either endogenous transporters or permeabilization. Such an approach has been used, e.g., for calibration of glucose sensors (Bittner et al., 2010), the lactate sensor Laconic (San Martín et al., 2013), or Peredox reporting the NADH/NAD^+^ ratio (Hung et al., 2011; Hung and Yellen, 2014; Köhler et al., 2018). These studies took advantage of the inherent permeability of the cells to either glucose, or lactate and pyruvate due to the expression of appropriate transporters. In contrast, cell membranes are not permeable for ATP and a plethora of reactions consume ATP within the cell. Nevertheless, also ATP sensors have recently been calibrated in organotypic brain slice cultures using permeabilization of cell membranes (Gerkau et al., 2019; Lerchundi et al., 2020). On the other hand, full calibration of nanosensors in readily accessible systems like purified proteins relies on the assumption that these calibration curves are valid also in cellular and/or more complex systems, but such approaches have successfully been used for obtaining quantitative information in various settings (e.g., San Martín et al., 2013; Fernández-Moncada and Barros, 2014; Mongeon et al., 2016). Furthermore, full calibration of metabolic nanosensors in combination with fluorescence life time imaging (FLIM) allows quantifying metabolite concentrations as, e.g., shown for Peredox, a sensor for the NADH/NAD^+^-redox ratio, or the glucose sensor SweetieTS (Mongeon et al., 2016; Díaz-García et al., 2017, 2019).

In summary, the dual nanosensor approach is a novel method for quantifying [ATP] from fluorescence data with its own advantages and limitations when compared to other methods (Table 1). Nevertheless, for certain applications like, e.g., complex and difficult to access tissues, it will provide an alternative/additional way for obtaining quantitative data.

### ATP in Astrocytes

The dual nanosensor approach was employed to determine the basal concentration of ATP in the cytosol of astrocytes both in primary cultures and acutely isolated brain slices. [ATP]_0_ was determined at around 1.5 mM for cultured cells (Figure 2) as well as between 0.7 mM and 1.3 mM for cortical astrocytes in brain slices (Figure 6). These values are well within the range of [ATP] reported for glial cells in culture (1.4 mM; Ainscow et al., 2002) as well as other cells like, e.g., neurons (1 mM to 4 mM; Fukuda et al., 1983; Ainscow et al., 2002; Mollajew et al., 2013; Rangaraju et al., 2014; Toloe et al., 2014; Pathak et al., 2015). However, the values are lower than data obtained from biochemical assays (3–7.5 mM; Schousboe et al., 1975; Silver and Erecinska, 1997; 10 mM calculated from ATP content: 40 nmol/mg protein, Winkler et al., 2017; and cytosolic volume of cultured astrocytes: 4.1 μl/mg protein, Dringen and Hamprecht, 1998). This difference is most likely due to the fact that nanosensors measure the concentration of free cytosolic ATP, while biochemical assays measure the total amount of ATP within a cell including organelles as well as ATP bound to proteins.

Based on the dual nanosensor approach, changes of [ATP] induced by the three different incubation conditions were calculated. Importantly, the resulting d[ATP] did not differ between the different methods of calculation (Figure 4). Furthermore, the values obtained using the calculation from each paired experiment, but also from the pooled data of all experiments yielded very similar results suggesting that this approach is robust against these experimental variables. In cultured cortical astrocytes, glutamate application induced a d[ATP] of −0.16 mM, amounting to about 11% of [ATP]_0_ (Figure 4), consistent with previous reports showing a (non-quantified) decrease in [ATP] (Magistretti and Chatton, 2005; Langer et al., 2017; Winkler et al., 2017). Uptake of glutamate released from synapses during neurotransmission is a major task for gray matter astrocytes (van den Berg et al., 1978; Bak et al., 2006) and the associated decrease in [ATP] has been implicated in the stimulation of astrocytic metabolism to support neighboring neurons (astrocyte-neuron-lactate shuttle hypothesis; Pellerin and Magistretti, 1994; Voutsinos-Porche et al., 2003).

Another sign of neuronal activity relevant for regulation of astrocytic metabolism is an increase of the concentration of extracellular K^+^ ([K^+^]_e_; Rash, 2010; Bittner et al., 2011; Ruminot et al., 2019). K^+^ is released from all neurons during repolarization and is, therefore, not restricted to glutamatergic neurons (Rash, 2010; MacVicar and Choi, 2017). An increase in [K^+^]_e_ induces glycogenolysis and activates astrocytic metabolism (Hof et al., 1988; Ruminot et al., 2011; Choi et al., 2012; MacVicar and Choi, 2017; Fernandez-Moncada et al., 2018; Köhler et al., 2018). Previously, an increase in the ATY signal induced by increasing [K^+^]_e_ from 3 mM to 8 mM or 12 mM was reported for astrocytes in culture and acutely isolated brain slices (Fernandez-Moncada et al., 2018; Lerchundi et al., 2019). Using the dual nanosensor approach and increasing [K^+^]_e_ from 5.4 mM to 12 mM, d[ATP] was quantified here as 0.07 mM or about 5% of [ATP]_0_ in cultured cortical astrocytes (Figure 4). The K^+^ induced increase of [ATP] is likely limited by the availability of free ADP within the cell and, therefore, the rather small increase in [ATP] might underestimate the level of activation of metabolism.

Finally, when blocking mitochondrial ATP production using azide, a decrease of [ATP] of about −0.5 mM (i.e., −32% of [ATP]_0_; Figure 4) with an initial rate of d[ATP] of −0.23 mM/min (Figure 5C) was observed, consistent with our previous biochemical measurements on the same culture preparations (Winkler et al., 2017). Astrocytes express all enzymes of glycolysis at rather high levels, but are also equipped with the enzymes necessary for fully oxidizing pyruvate and generating ATP in mitochondria (Lovatt et al., 2007). Nevertheless, astrocytes tolerate inhibition of mitochondrial ATP production well by upregulating glycolysis resulting in an increased NADH/NAD^+^-redox ratio and increased lactate production (Dringen et al., 1993; Bittner et al., 2010; Wilhelm and Hirrlinger, 2011; San Martín et al., 2013; Supplie et al., 2017; Westhaus et al., 2017), consistent with the surprising finding that mice lacking functional mitochondria in astrocytes survive for more than a year without any phenotype (Supplie et al., 2017). However, because numerous mechanisms can contribute to an increase of the glycolytic rate including, e.g., upregulation of glucose transport, upregulation of glycolytic enzymes or activation by allosteric regulators like, e.g., fructose-2,6-bisphosphate, the precise mechanism remains to be elucidated.

In summary, the dual nanosensor approach allows estimating the basal concentration of a metabolite of interest (here ATP) based on the signal changes of two nanosensors with different equilibrium constants. It adds an option to the toolbox for quantifying changes in cellular metabolite concentrations from fluorescence changes, which is particularly useful if determination of R_max_ is difficult or impeded since the dual sensor approach is independent of this parameter. Finally, this method is not only applicable to metabolic nanosensors, but also for other sensors reporting, e.g., pH or the concentration of ions or second messengers (San Martin et al., 2014; Zhang et al., 2018; Bischof et al., 2019; Depaoli et al., 2019).

## Data Availability Statement

All datasets presented in this study are included in the article/Supplementary Material.

## Ethics Statement

The animal study was reviewed and approved by Animal Welfare Office of the Faculty of Medicine, University of Leipzig and Landesdirektion Sachsen.

## Author Contributions

JH conceptualized and designed the study. SK, PF, and UW acquired data. SK, PF, HS, JH, and UW analyzed and interpreted the data. HS performed analysis using Mathematica. JH and UW supervised the project. JH acquired funding. SK, JH, and UW wrote the manuscript. All authors contributed to manuscript revision, read, and approved the submitted version.

## Conflict of Interest

The authors declare that the research was conducted in the absence of any commercial or financial relationships that could be construed as a potential conflict of interest.
