# Supplementary material for: A Dual Nanosensor Approach to Determine the Cytosolic Concentration of ATP in Astrocytes
Source: Front Cell Neurosci. 2020 Sep 18;14:565921. doi: 10.3389/fncel.2020.565921 (PMC7530325; doi:10.3389/fncel.2020.565921)
Supplement: Supplementary file 1 [file Data_Sheet_1.PDF]

Supplementary Material

A dual nanosensor approach to determine the cytosolic concentration of ATP in astrocytes

Susanne Köhler[1], Hartmut Schmidt[1], Paula Fülle[1,2], Johannes Hirrlinger[1,3,*], Ulrike Winkler[1]

[1]Carl-Ludwig-Institute for Physiology, Faculty of Medicine, University of Leipzig, Liebigstr. 27, D-04103 Leipzig, Germany

[2]Wilhelm-Ostwald-Schule, Gymnasium der Stadt Leipzig, Willi-Bredel-Straße 15, 04279 Leipzig, Germany

[3]Department of Neurogenetics, Max-Planck-Institute for Experimental Medicine, Hermann-Rein-Str. 3, D-37075 Göttingen, Germany

Supplementary Figure

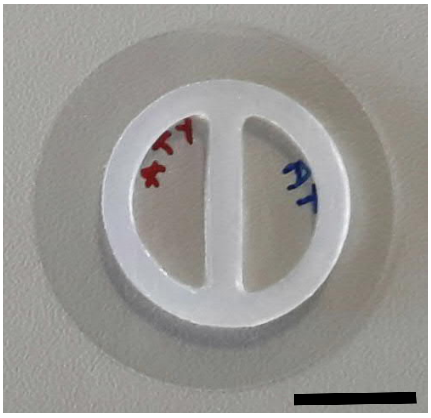

Supplementary Figure 1. Custom made silicon ring with two incubation chambers attached to a coverslip (30 mm diameter). Rings are mounted on the coverslip and autoclaved, and placed in 6-well cell culture plates prior to seeding the cells. For transfection, medium is removed and transfection medium is applied to both chambers separately. Prior to imaging, the rings are carefully removed allowing flow of the perfusion medium to cells transfected with different plasmids simultaneously. Scale bar: 1 cm.

Supplementary Table 1. Variables and indices used in the equations

**A: Variables**	
I_{FRET}	fluorescence intensity recorded in the FRET channel
I_{CFP}	fluorescence intensity recorded in the CFP channel
I	I_{FRET} / I_{CFP}
[ATP]	cytosolic concentration of ATP
R	fluorescence signal of the sensor<br>normalized to R_{min} (set as 0) and R_0 (set as 1)<br>as defined in eq. 1
dR	relative change of R. $dR = R / R_0 - 1$
B	sensor occupancy (= ATP-bound sensor / total ATP sensor)
n_H	Hill-coefficient
k_D	dissociation constant

**B: Indices**	
0	parameter at baseline conditions
min	parameter at [ATP] = 0
max	parameter at saturation of the sensor
treat	parameter during treatment of the cells<br>(i.e. application of azide, glutamate or high [K^+])
AT	ATeam1.03
ATY	ATeam1.03YEMK
AT^{MUT}	$AT1.03^{R122K/R126K}$
